# Rapid and multiplex preparation of engineered *Mycobacterium smegmatis* porin A (MspA) nanopores for single molecule sensing and sequencing†

**DOI:** 10.1039/d1sc01399h

**Published:** 2021-06-08

**Authors:** Shuanghong Yan, Liying Wang, Xiaoyu Du, Shanyu Zhang, Sha Wang, Jiao Cao, Jinyue Zhang, Wendong Jia, Yuqin Wang, Panke Zhang, Hong-Yuan Chen, Shuo Huang

**Affiliations:** State Key Laboratory of Analytical Chemistry for Life Sciences, School of Chemistry and Chemical Engineering, Nanjing University 210023 Nanjing China shuo.huang@nju.edu.cn; Chemistry and Biomedicine Innovation Center (ChemBIC), Nanjing University 210023 Nanjing China

## Abstract

Acknowledging its unique conical lumen structure, *Mycobacterium smegmatis* porin A (MspA) was the first type of nanopore that has successfully sequenced DNA. Recent developments of nanopore single molecule chemistry have also suggested MspA to be an optimum single molecule reactor. However, further investigations with this approach require heavy mutagenesis which is labor intensive and requires high end instruments for purifications. We here demonstrate an efficient and economic protocol which performs rapid and multiplex preparation of a variety of MspA mutants. The prepared MspA mutants were demonstrated in operations such as nanopore insertion, sequencing, optical single channel recording (oSCR), nanopore single molecule chemistry and nanopore rectification. The performance is no different from that of pores however prepared by other means. The time of all human operations and the cost for a single batch of preparation have been minimized to 40 min and 0.4$, respectively. This method is extremely useful in the screening of new MspA mutants, which has an urgent requirement in further investigations of new MspA nanoreactors. Its low cost and simplicity also enable efficient preparations of MspA nanopores for both industrial manufacturing and academic research.

## Abstract 2

A rapid and multiplex approach to prepare engineered *Mycobacterium smegmatis* porin A (MspA) nanopores for single molecule sensing and sequencing.

## Graphical abstract

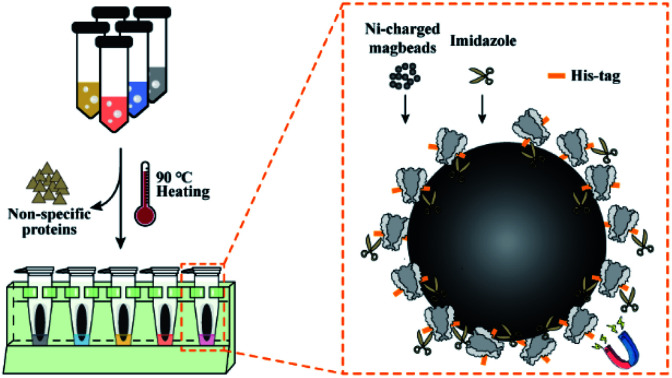

## 1 Introduction

*Mycobacterium smegmatis* porin A (MspA),^1^ the major porin of *Mycobacterium smegmatis*,^2^ is a conical shaped pore forming protein composed of eight tightly interconnected monomeric subunits, measuring ∼20 kDa for each component.^3^ Acknowledging its unique lumen structure, MspA possesses a high spatial resolution, which is capable of resolving substitutions of a single base in a strand of DNA.^4^ Its overall structure is composed of rigid β-barrels, which minimize measurement noises when applied as a nanopore. These features have enabled the first demonstration of nanopore sequencing of DNA^5,6^ and applications thereof, such as epigenetic sequencing,^7^ miRNA sequencing,^8^ DNA lesion sequencing^9^ or nanopore tweezers.^10^

Engineered biological nanopores may also be applied as a nanoreactor,^11^ a concept pioneered by Hagan Bayley's group since 1997 ^12^ and demonstrated by a variety of engineered alpha-hemolysin mutants thereafter.^13–18^ Though these specially designed nanoreactors are capable of resolving formation and breaking of an individual chemical bond, the reported event amplitudes are generally low, indicating a limited resolution to further resolve more refined chemical processes. MspAs well engineered as nanoreactors,^19,20^ with a much-enlarged event amplitude, were reported, acknowledging its conical structure which serves to amplify subtle perturbations caused by single molecule reactions happening in the vicinity of the pore constriction. To further develop this technique, there is an urgent need for significant effort in the screening of a variety of heavily mutated MspAs.^20^ However, to date, there is no report of a method that rapidly prepares MspA mutants in a highly multiplexed manner so that a variety of MspA mutants may be prepared in parallel.

According to reported literature studies, MspA was prepared by whole cell extraction with organic solvents directly from cultured *Mycobacterium smegmatis* strains.^2^ However, this strategy reports a low yield and may include interferences from other non-desired channel proteins during downstream nanopore measurements. As a recombinant protein, MspA was also expressed by *E. coli* and subsequently purified by anion exchange chromatography^21^ or nickel affinity chromatography.^19,20^ However this strategy requires the use of fast protein liquid chromatography (FPLC) instruments for purification. FPLC has an extremely low throughput and which requires significant human labor when a large variety of different MspA mutants are to be prepared. An FPLC instrument, such as a GE AKTA PURE (General Electronics, USA) costs $40 000 for a base model and its operation requires a significant amount of training for non-specialists. Considering that prepared MspA octamers can't be obtained from any commercial vendors, a small academic group which may be interested in this pore has to seek collaborations with other groups with a relevant research background, forming a technical hurdle for most research groups.

It has been previously reported that wild type (WT) MspA is stable against temperatures up to 100 °C.^22^ This unique thermal stability has assisted in the selective extraction of WT MspA directly from *Mycobacterium smegmatis* lysates. However, further purifications with anion exchange chromatography and size exclusion chromatography were still required to reach the desired purity.^23^ Acknowledging the thermal stability of MspA, we demonstrate a rapid and multiplexed protocol aiming to prepare a large variety of MspA mutants in parallel. All prepared MspA mutants were tested in corresponding downstream single molecule measurements and their performance is no different from that of pores however prepared by other means.^23,24^ This protocol doesn't require any high-end instruments and the time of human operation has been minimized. It is extremely useful in the screening of functional MspA mutants, aiming for different single molecule chemistry applications. It is also attractive for small academic groups that are interested in applications of MspA but may be short of budget to maintain an FPLC or lack the desired expertise. For technology validation and exchanging, all plasmid DNAs used in this work are openly shared in the Molecular Cloud repository (https://www.molecularcloud.org/s/shuo-huang) (ESI Methods 1†).

## 2 Results and discussion

### 2.1. An overview of the methodology

A variety of MspA mutants have been developed respectively serving as a sensor,^1,25^ a sequencer^5,6^ or a single molecule reactor.^19,20,26^ In preparation of MspA and its mutants thereof, liquid chromatography techniques were widely applied, which however require an expensive FPLC instrument and corresponding columns for a desired performance. Though proteins purified by an FPLC generally demonstrate highly desired purity, its low throughput is however a limiting factor when a large variety of proteins are to be purified.

We present here a newly designed protocol which significantly reduces the cost and the complexity of MspA preparation, as all operations can now be carried out in parallel. Considering that the C-terminus of MspA is located on the outer surface of the pore vestibule,^3^ the addition of a hexa-histidine tag (His-tag) does not interfere with the assembly and the sensing feature of MspA. This has been experimentally approved in previous studies that we have reported.^19,24^ Acknowledging the added His-tag, purification of MspA could be carried out using Ni-charged magnetic beads so that an FPLC can be avoided. Though the MspA nanopore purified by Ni-charged magnetic beads may still contain contaminant proteins, it is not critical as long as the contaminant proteins don't insert themselves into the membrane as an MspA does. Acknowledging the thermal stability of MspA, the resuspended cells containing the MspA nanopores were thoroughly heated prior to purification, which efficiently lyses the cell to improve the yield and thus a sonication instrument is well omitted. Thermal treatment also efficiently eliminates contaminant proteins that are however not thermally stable.

The thermal stability of M2 MspA was tested as described in Fig. S1,† which showed that thermal incubation of M2 MspA at 90 °C will not damage its octameric pore assembly. We thus tested our protocol with M2 MspA for a feasibility test (ESI Methods 2†). The preparation was monitored in a stepwise manner and characterized by gel electrophoresis (Fig. S2†), which clearly indicated that thermal treatment accompanied by magnetic separation has successfully produced MspA mutants in the desired octameric form. Without any further purification, the prepared M2 MspA was directly applied in single channel recordings (Fig. S3, ESI Methods 3†). Spontaneous channel insertions were sequentially observed and the measured open pore currents were uniformly distributed, indicating a high consistency of channel conductance (Fig. S2B and C†). In addition to that, the open pore currents at a +20 mV applied potential obtained by this method and conventional methods were almost identical (Fig. S4†), which concludes that the nanopores prepared by this method are no different from those prepared by conventional methods. The thermal stability of M2 MspA is also retained during single channel recording. Experimentally, by modulating the measurement temperature between 4 °C and 50 °C on an Orbit Mini apparatus (Nanion Technologies, Germany), we observed that the pore stayed open (Fig. S5†). Though the built-in temperature control module of Orbit Mini is restricted to a limited temperature range, it nevertheless suggests the feasibility of measurements with MspA at an extreme temperature up to 90 °C.

**Fig. 1:**
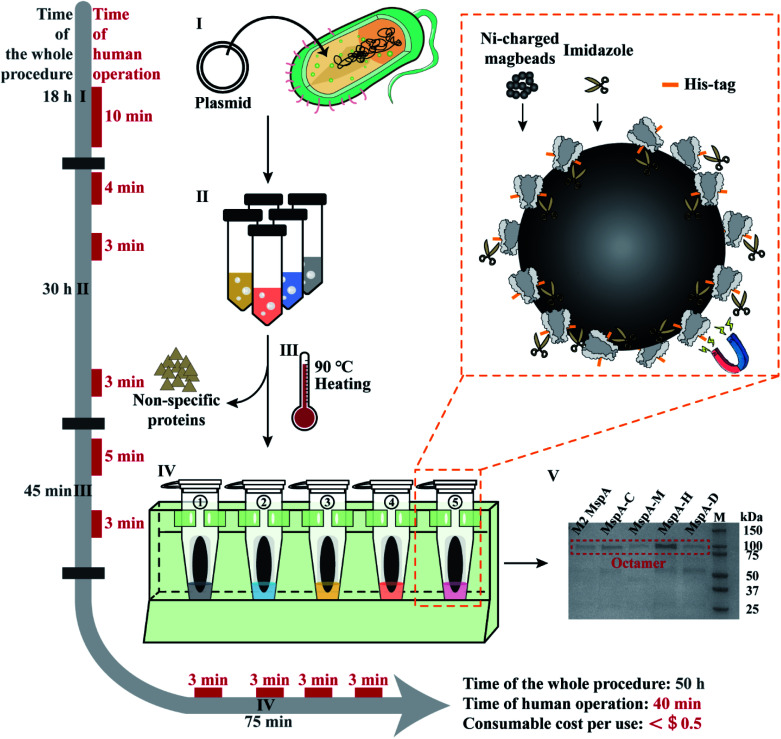
The workflow of rapid and multiplex MspA preparation. Phase I: the plasmid DNAs encoding for MspA mutants (Table S2†) were heat-shock transformed into *E. coli* BL21(DE3) competent cells and cultured on an agar plate. Human operations: agar plate preparation and heat-shock transformation (10 min). Phase II: single colonies collected from phase I are grown in liquid LB medium, IPTG induced and cultured overnight. The cells were harvested by centrifugation. Human operations: single colony collection (4 min), IPTG induction (3 min) and bacteria pellet collection (3 min). Phase III: the collected pellets are resuspended in the lysis buffer and heated at 90 °C for 10 min in a heating block to thoroughly lyse the cells. A significant portion of non-specific proteins was fully denatured during heating. Centrifugation was performed to collect the supernatant which contains the desired proteins. Human operations include lysis buffer exchange and pellet resuspension (5 min) along with supernatant collection (3 min). Phase IV: the supernatant of the bacterial lysate is purified using Ni-charged magbeads (the orange dashed box). Briefly, the beads are incubated with the supernatant and sequentially washed with washing buffer A, eluting buffer B1 and eluting buffer B2. Human operations include adding the supernatant of bacterial lysate to tubes (3 min), adding washing buffer A to tubes (3 min), adding eluting buffer B1 to tubes (3 min) and adding eluting buffer B2 to tubes (3 min). Phase V: gel electrophoresis diagram for the preparation of 5 kinds of MspA nanopores simultaneously. Gel electrophoresis was carried out with a 4–20% Mini-PROTEAN TGX Gel (Cat. #4561083, Bio-Rad) and a 200 V bias was applied for 27 min. Lane M: precision plus protein standards (Bio-Rad). All procedures such as plasmid transformation, liquid culturing, lysate heating and magnetic separation can be carried out in parallel. The time of human operations was only 40 min in total. The preparation doesn't require any high-end instruments and the consumable costs are negligible.

Based on the results of this feasibility test, this protocol was further adapted to prepare a variety of different MspA mutants in parallel. The general workflow is described in Fig. 1 and detailed in Fig. S6.† Briefly, 100 ng plasmid DNAs encoding five different MspA mutants (M2 MspA, MspA–C, MspA–H, MspA–M and MspA–D) were respectively heat-shock transformed into 100 μL *E. coli* BL21 (DE3) competent cells (Biomed). The competent cells and plasmid DNA were respectively mixed in 1.5 mL tubes and gently tapped. Then the tubes were ice incubated for 30 min. The tubes were then incubated at 42 °C for 90 s in a metal heating block and ice incubated for another 3 min. The transformed cells were spread evenly on the surface of different agar plates (0.76 g LB agar with 20 mL Milli-Q water for each plate) and incubated at 37 °C for 18 h.

The supernatants of different bacterial lysates were respectively added to the pre-treated beads (ESI Methods 4†) and shaken on a rotary mixer at room temperature (RT) for 60 min. After magnetic separation, the supernatant in each tube was discarded. Then 1 mL washing buffer A (0.5 M NaCl, 20 mM HEPES, 5 mM imidazole, 0.5% (w/v) Genapol X-80, pH = 8.0) was added to the beads in each tube. The beads were resuspended and shaken on a rotary mixer at room temperature (RT) for 5 min. After magnetic separation in parallel, the supernatant in each tube was discarded. Then 100 μL eluting buffer B1 (0.5 M NaCl, 20 mM HEPES, 219.5 mM imidazole, 0.5% (w/v) Genapol X-80, pH = 8.0) was added to the beads in each tube. The beads were resuspended and shaken on a rotary mixer at room temperature (RT) for another 5 min. After magnetic separation, the supernatant in each tube was discarded. Eventually, 100 μL eluting buffer B2 (0.5 M NaCl, 20 mM HEPES, 500 mM imidazole, 0.5% (w/v) Genapol X-80, pH = 8.0) was added to the beads in each tube. The tubes were shaken on a rotary mixer at room temperature (RT) for 5 min. After magnetic separation, the supernatant in each tube was collected. As observed from the characterization results of gel electrophoresis on a 4–20% Mini-PROTEAN TGX Gel (Cat. #4561083, Bio-Rad) (Fig. 1), the collected supernatant contained the desired MspA mutants in an octameric form. The detailed purification steps are shown in Fig. S7.† Though noticeable traces of non-desired proteins were still seen on the gel, they did not interfere in any downstream nanopore measurements since spontaneous insertions of these proteins were not observed during continuous single channel recordings (Fig. S8–S11†). All five types of nanopores were directly used without any further purifications. To minimize the cost of consumables, Ni-charged magbeads can be regenerated for multiple uses (ESI Methods 5†).

All experimental procedures such as plasmid transformation, bacterial culturing, lysate heating and magnetic separation were designed in such a way that they can be carried out in parallel. Different from reported strategies of MspA preparation,^1,24^ this approach is extremely simplified and can be simultaneously carried out for different MspA mutants. The preparation doesn't require any chromatography instruments and the consumable costs have been minimized, being ∼$0.4 per single mutant for each preparation, based on a rough estimation (ESI Methods 6†). Though the whole procedure of preparation takes ∼50 h for this protocol, the time of human operations is only 40 min in total.

### 2.2. Applications of prepared nanopores

**Fig. 2:**
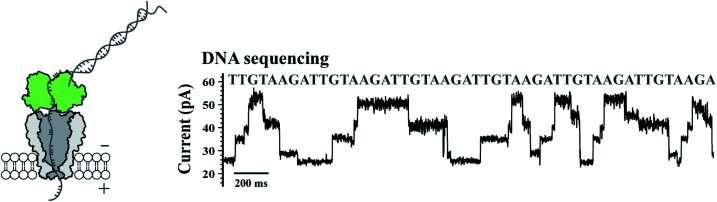
Application of M2 MspA in DNA sequencing. The schematic diagram (left) and a representative trace of nanopore sequencing of DNA (right). Nanopore sequencing was performed as previously published.^9^ The measurements were performed in a 0.3 M KCl buffer (0.3 M KCl, 10 mM MgCl_2_, 10 mM (NH_4_)_2_SO_4_, 4 mM DTT and 10 mM HEPES, pH = 7.5) buffer. A +180 mV potential was continuously applied. A DNA template, primer and blocker (Table S1†) were designed for a simple demonstration of nanopore sequencing. Phi29 DNA polymerase (green, left diagram) was applied as the molecular motor during nanopore sequencing. M2 MspA (grey, left diagram) applied for these measurements was from the corresponding sample as demonstrated in the gel results of Fig. 1.

To validate their single molecule sensing performance, the simultaneously prepared MspA mutants were respectively tested in corresponding single molecule measurements. M2 MspA, which is known to sequence DNA,^5^ was tested in a nanopore sequencing assay, in which a synthetic single stranded DNA, namely the template (Table S1†), was sequenced. The template strand was hybridized with the primer and the blocker strand (Table S1†) to form a sequencing library complex, which is to be read by the subsequent nanopore sequencing assay. The template strand containing a segment of six sequence repeats of AGAATGTT (5′-3′) was designed. During nanopore sequencing, the sequence of AGAA reports the highest blockage level and the sequence of TGTT reports the lowest. It is expected that alternate reading between AGAA and TGTT reports triangle shaped nanopore sequencing patterns. The assay was carried out similarly to that described previously^9^ and demonstrated with more details in Fig. 2. A phi29 DNA polymerase was applied as the motor protein to drag the template through the pore constriction in a stepwise manner. According to nanopore sequencing results, six triangle-shaped nanopore sequencing patterns were clearly observed, indicating that the prepared M2 MspA (Fig. 1) can be directly applied to perform nanopore sequencing. Though not demonstrated in this paper, M2 MspA prepared by this protocol is in principle suitable for any nanopore sequencing based single molecule assay that has been previously carried out using M2 MspA. These assays include but not limited to sequencing of DNA,^5,6^ epigenetic markers,^7^ xeno nucleic acid,^24^ miRNA,^8^ DNA lesions^9^ or nanopore sequencing based single molecule enzymology assays.^10^

**Fig. 3:**
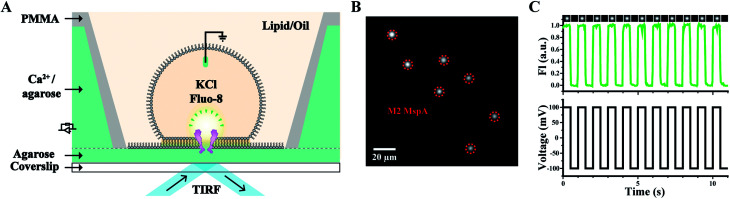
Microscopic imaging of M2 MspA. (A) The schematic diagram of the measurement device. A DIB was formed between the droplet and the hydrogel substrate. M2 MspA spontaneously inserts itself into the DIB, forming channels connecting the electrolyte in the droplet and the hydrogel substrate. Transport of calcium ions from the substrate to the droplet results in the appearance of fluorescence spots.^27^ A positively applied bias enhances the transport of calcium ions which results in an enhanced fluorescence intensity. (B) A frame containing multiple fluorescence spots representing inserted M2 MspA nanopores (red circles) in the DIB. Scale bar: 20 μm. (C) A representative fluorescence-time trace. A square wave voltage protocol was applied. The fluorescence intensity (FI) change was modulated by the voltage protocol indicating that the fluorescent spot results from inserted channel proteins. Cropped images containing a single pore at different frames of the measurements were placed on top of the fluorescence-time trace. The measurements were performed as described in ESI Methods.† A buffer combination of 1.5 M KCl buffer (*cis*, droplet), and 0.75 M CaCl_2_ buffer (*trans*, hydrogel substrate) was applied during the measurements. The M2 MspA applied for these measurements was from the corresponding sample as demonstrated in the gel results of Fig. 1.

Other than nanopore sequencing, the prepared M2 MspA was also validated by oSCR (ESI Methods 7†). The measurements were carried out by fluorescence imaging of droplet interface bilayers (DIBs) by total internal reflection fluorescence (TIRF) microscopy, similarly to that previously described.^27^ The schematic diagram of the DIB device is shown in Fig. 3A. Briefly, a coverslip coated with agarose (1.0% w/v in Milli-Q water) adhered to the bottom of the measurement device. Molten agarose (2.5% w/v in 0.75 M CaCl_2_, 10 mM HEPES, pH = 7.0) was filled into the device. In the lipid/oil environment (5 mg mL^−1^ DPhPC in the mixture of hexadecane/silicone oil with a 1 : 1 volume ratio), a semi-permeable membrane was formed between a droplet (consisting of 1.5 M KCl, 400 μM EDTA, 33 μM Fluo-8, 10 mM HEPES, pH = 7.0 and M2 MspA) and the agarose substrate. The compartment in the droplet is defined as the *cis* side and the hydrogel substrate filled with CaCl_2_ is defined as the *trans* side. Once a nanopore was inserted into the DIB, Ca^2+^ at the *trans* side migrates through the pore and binds to Fluo-8 at the *cis* side, generating a bright fluorescence spot, as marked by the red dashed circles of Fig. 3B. To validate that the fluorescence spot results from an inserted pore instead of a statically fluorescing contaminant, a square-wave (1 Hz, ±100 mV) voltage protocol was applied. Experimentally, the fluorescence intensity of the spots was observed to be periodically fluctuating, synchronized with the applied square-wave protocol (Movie S1†). The corresponding fluorescence trace of M2 MspA was as well demonstrated (Fig. 3C). Though highly simplified, the above demonstration has validated that the prepared M2 MspA is directly compatible with an oSCR assay. Though not demonstrated here, the same principle can be applied to other MspA mutants in an oSCR^27^ or a DiffusiOptoPhysiology (DOP) assay^28^ as well.

**Fig. 4:**
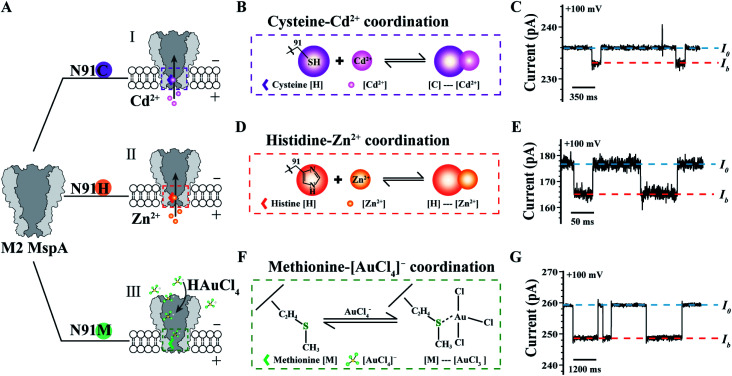
Single molecule chemistry reactions observed with different MspA mutants. (A) The amino acid at site 91 (Asparagine, N) of an M2 MspA nanopore was respectively engineered to cysteine (I)/histidine (II)/methionine (III) to form different MspA mutants. The mutants were treated as single molecule chemical reactors, which are sensitive to binding from different metal ions. (B) The schematic diagram of the coordination chemistry between a cysteine and Cd^2+^. (C) A representative trace containing Cd^2+^ binding events to an MspA–C. (D) The schematic diagram of the coordination chemistry between a histidine and Zn^2+^. (E) A representative trace of containing Zn^2+^ binding events to an MspA–H. (F) The schematic diagram of the coordination chemistry between a methionine and a [AuCl_4_]^−^. (G) A representative trace containing [AuCl_4_]^−^ binding events to an MspA–M. *I*_0_ refers to the open pore current and *I*_b_ refers to the state when an analyte was bound to the pore (C, E and G). The measurements for MspA–C (B and C) were performed in 1 M NaCl buffer (1 M NaCl, 10 mM HEPES, 0.4 mM TCEP, pH = 7.4). The measurements for MspA–H (D and E) were performed in 1 M NaCl buffer (1 M NaCl, 10 mM HEPES, pH = 7.4). The measurements for MspA–M (F and G) were performed in 1.5 M KCl buffer (1.5 M KCl, 10 mM Tris–HCl, pH = 7.0). Cd^2+^ (B and C) or Zn^2+^ (D and E) was placed in *trans* with a 6 μM or 1 μM concentration. [AuCl_4_]^−^ (F and G) was placed in *cis* with a 1 μM concentration. All measurements were performed as described in ESI Methods.† A +100 mV bias was continuously applied. All MspA mutants applied for measurements in B–G were from the corresponding sample as demonstrated in the gel results of Fig. 1.

The MspA mutants simultaneously prepared by this protocol can as well be applied as single molecule nanoreactors, of which the amino-acid residues around the pore constriction become critical by participating with the single molecule reactions to be observed. To be specific, the MspA–C,^20^ MspA–H^20^ and MspA–M^19^ mutants were designed which respectively replace the original asparagine at site 91 of an M2 MspA with a cysteine, a histidine or a methionine (Fig. 4A). Experimental observations of single molecule chemical reactions with MspA–C, MspA–H and MspA–M have been previously performed. To demonstrate that MspA mutants simultaneously prepared by this protocol can be equivalently used, corresponding measurements were respectively carried out with MspA–C, MspA–H or MspA–M similar to that previously reported.^19,20^ All measurements were carried out as described in the ESI Methods† and Fig. 4. A +100 mV bias was continuously applied and binding of an analyte to the pore results in clear resistive pulses observed during single channel recording. Specifically, the coordination reaction between a cysteine and a Cd^2+^ or between a histidine and a Zn^2+^ was respectively observed with MspA–C or MspA–H (Fig. 4B–E). A tetrachloroaurate (III) ([AuCl_4_]^−^), which is a polyatomic anion with a central atom of gold, preferentially reacts with sulfur containing amino acid residues such as a methionine. This reaction has thus been observed with MspA–M (Fig. 4F and G). Statistics of blockage events of MspA–M (Table S3†) were identical to those reported previously however with MspA–M prepared by conventional methods,^19^ confirming that the event feature is not changed with nanopores prepared by this new method.

**Fig. 5:**
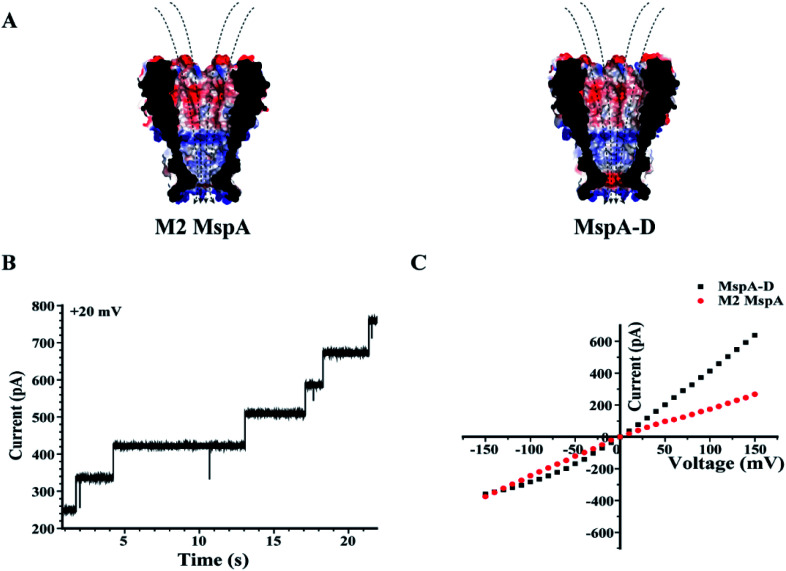
The rectification properties of M2 MspA and MspA–D. (A) The charge distributions of M2 MspA and MspA–D. The amino acid at site 91 of M2 MspA or MspA–D is asparagine or aspartic acid, which differs in charge when measured in a buffer of a neutral pH. In an octameric pore assembly, eight amino acid residues were identically altered. When compared with an octameric M2 MspA, the MspA–D mutant introduces a ring of eight negatively charged residues at the pore constriction. (B) Continuously observed spontaneous insertion of MspA–D during single channel recording. A +20 mV bias was continuously applied. The appearance of equally spaced current steps indicates that the octameric MspA–D is active for pore insertion. (C) Representative *I*–*V* curves acquired from M2 MspA and MspA–D. The measurements were performed in a 1 M KCl buffer (1 M KCl, 10 mM HEPES, pH = 7.0). A voltage ramp was scanned from −150 mV to +150 mV to derive the *I*–*V* curve. Generally, different rectification behaviours were observed between these two types of pores. The introduced negative charges in MspA–D have significantly enhanced the channel conductance at a positively applied potential. The M2 MspA and MspA–D applied for measurements in B and C were from the eluent as demonstrated in the gel results of Fig. 1.

Site 91 of an M2 MspA can as well be replaced with an aspartic acid to generate the MspA–D mutant^20^ (Fig. 5). Though the MspA–D hardly reacts with any of the ions discussed above, the introduced negative charge near the pore constriction significantly modulates the open pore conductance and its rectification behaviour. This has been demonstrated by a comparison of representative *I*–*V* curves between an M2 MspA and an MspA–D (Fig. 5C), with which a simple charge modulation by replacement of a single amino acid has resulted in a dramatic change of the ion transport properties of the channel.

To summarize, all MspA mutants obtained by this rapid, simplified and multiplexed preparation method were tested to check if they meet the need of single molecule measurements. The yield of protein production is also evaluated to be no different from that of conventional methods.^29^ In a side by side comparison with a 100 mL culture volume, 59 μg M2 MspA was produced using this method and a 56 μg yield was produced by conventional methods, demonstrating a similar total yield of both methods. This protocol may as well be applied to rapid preparation of other nanopores to which a His-tag can be added and a similar thermal stability is shown. We however admit that the demonstrated method is not generally suitable for proteins that don't simultaneously meet these two criteria. Nevertheless, this method is particularly useful in simultaneous preparations of a variety of MspA mutants with a known sensing feature or be applied as a screening assay in the search for MspA mutants with new sensing properties. There is a possibility that some MspA mutants may lose their thermal stability which may reduce the yield when prepared by this method. However, based on our experience with more than 10 engineered MspAs with mutations around the pore constriction, this thermal stability seems to be highly conserved. Based on previous studies of the crystal structure of MspA,^30^ it is possible to engineer any site of this protein. However, it is hard to conclude whether any mutation point is needed for nanopore assembly in the lipid membrane or predict whether any mutagenesis made to a particular site would interfere with nanopore assembly. Biophysics wise, we believe that nanopore assembly is a cooperative process involving more than one amino acid. It is however fortunate that the multiplex nature of this preparation method is suitable to screen a large variety of nanopore mutants with ease.

## 3 Conclusions

In summary, we have demonstrated a highly simplified protocol, which is specially designed to fit an urgent upcoming need for rapid and multiplexed preparation of a variety of MspA mutants. The prepared nanopores have been tested in operations such as nanopore insertion, nanopore sequencing and oSCR. Some MspA mutants were also applied as specific nanoreactors to observe single molecule chemical reactions. All the above demonstrations indicate that the prepared MspA mutants are no different from those prepared as previously reported. The prepared pores can be directly applied in any nanopore sequencing related application. These include but are not limited to sequencing of DNA,^5,6^ epigenetic modifications,^7^ miRNA,^8^ DNA lesion^9^ or enzymatic kinetics.^10^ The costs of labour and reagents are however negligible and the investment of an FPLC has been omitted. This method is in principle suitable to prepare other protein nanopores that show a similar thermal stability. It is however particularly suitable in the screening of MspA mutants for observations of desired single molecule chemical reactions, in which the multiplexity of this method is advantageous. These nanoreactor pores may be applied to probe the presence of metal ions or small molecular analytes with a high resolution. The simplicity and low cost of the methodology are also ideal for industrial preparations of MspA mutants or for small academic groups who have a limited budget for investment in nanopore preparations.

## Author contributions

S. H. Y., L. Y. W. and S. H. conceived the project. L. Y. W., S. H. Y., S. Y. Z., and X. Y. D. prepared the MspA nanopores. L. Y. W. performed the measurements. J. C., S. W., J. Y. Z., and W. D. J. provided inspiring discussions. P. K. Z. and Y. Q. W. set up the instruments. H.-Y. C. and S. H. supervised the project.

## Data availability

All data presented in this work can be provided by the corresponding authors upon reasonable requests.

## Conflicts of interest

There are no conflicts to declare.

## Supplementary Material

SC-012-D1SC01399H-s001

SC-012-D1SC01399H-s002
