# Supplementary material for: Rapid and multiplex preparation of engineered Mycobacterium smegmatis porin A (MspA) nanopores for single molecule sensing and sequencing
Source: Chem Sci. 2021 Jun 8;12(27):9339–46. doi: 10.1039/d1sc01399h (PMC8278974; doi:10.1039/d1sc01399h)
Supplement: SC-012-D1SC01399H-s001 [file SC-012-D1SC01399H-s001.pdf]

Electronic Supplementary Material (ESI) for Chemical Science.
This journal is © The Royal Society of Chemistry 2021

Supporting Information (SI)

Rapid and multiplex preparation of engineered *Mycobacterium smegmatis* porin A (MspA) nanopores for single molecule sensing and sequencing

Shuanghong Yan,[#ab] Liying Wang,[#ab] Xiaoyu Du,[ab] Shanyu Zhang,[ab] Sha Wang,[ab] Jiao Cao,[ab] Jinyue Zhang,[ab] Wendong Jia,[ab] Yuqin Wang,[ab] Panke Zhang,[ab] Hong-Yuan Chen,[ab] and Shuo Huang*[ab]

[a.] State Key Laboratory of Analytical Chemistry for Life Sciences, School of Chemistry and Chemical Engineering, Nanjing University, 210023, Nanjing, China.

[b.] Chemistry and Biomedicine Innovation Center (ChemBIC), Nanjing University, 210023, Nanjing, China.

[#] These authors contributed equally to this work.

[*]Corresponding author. Email: shuo.huang@nju.edu.cn (S.H.)

Materials

Ni-charged magnetic beads were purchased from Genscript (New Jersey, USA). 1,2-diphytanoyl-sn-glycero-3-phosphocholine (DPhPC) was from Avanti Polar Lipids. Pentane, hexadecane, tris(2-carboxyethyl) phosphine hydrochloride (TCEP), ethylenediaminetetraacetic acid (EDTA), hydrogen tetrachloroaurate (III) hydrate (99.99%) and Genapol X-80 were from Sigma-Aldrich. Potassium chloride, calcium chloride, sodium chloride (99.99%), zinc sulfate heptahydrate (99.995%), cadmium sulfate, 8/3-hydrate (99.99%), sodium hydroxide (99.9%), sodium hydrogen phosphate and sodium dihydrogen phosphate were from Aladdin (China). Hydrochloric acid (HCl) was from Sinopharm (China). 4-(2-hydroxyethyl)-1-piperazine ethanesulfonic acid (HEPES) was from Shanghai Yuanye Bio-Technology (China). Dioxane-free isopropyl-β-D-thiogalactopyranoside (IPTG), kanamycin sulfate, imidazole, DL-Dithiothreitol (DTT) and tris (hydroxymethyl)aminomethane (Tris) were from Solarbio. Phi29 DNA polymerase (phi29 DNAP) and dNTPs were purchased from New England Biolabs. DNA oligonucleotides (Table S1) were purchased from Genscript (New Jersey, USA). SDS-PAGE electrophoresis buffer powder was from Beyotime (China). Chelex 100 chelating resin (biotechnology grade, 100-200 mesh, sodium form), Precision Plus ProteinTM Dual color Standards and TGXTM FastCastTM Acylamide Kit (4-20%) were from Bio-rad. *E. coli* strain BL21(DE3) was from BioMed (China). LB broth and LB agar were from Hopebio (China). All the items listed above were used as received.

1M KCl (1M KCl, 10 mM HEPES, pH=7.0), 0.3 M KCl buffer (0.3 M KCl, 10 mM $MgCl_2$, 10 mM $(NH_4)_2SO_4$, 4 mM DTT and 10 mM HEPES, pH=7.5), 1.5 M KCl buffer (1.5 M KCl, 10 mM Tris-HCl, pH=7.0), 1 M NaCl buffer (1 M NaCl, 10 mM HEPES, pH=7.4), lysis buffer (100 mM Na_2HPO_4/NaH_2PO_4, 0.1 mM EDTA, 150 mM NaCl, 0.5% (w/v) Genapol X-80, pH=6.5), LE buffer (50 mM NaH_2PO_4, 300 mM NaCl, pH=8.0), buffer A (0.5 M NaCl，20 mM HEPES，5 mM Imidazole，0.5% (w/v) Genapol X-80，pH=8.0), buffer B1 (0.5 M NaCl，20 mM HEPES，219.5 mM Imidazole，0.5% (w/v) Genapol X-80，pH=8.0) and buffer B2 (0.5 M NaCl，20 mM HEPES，500 mM Imidazole，0.5% (w/v) Genapol X-80，pH=8.0) were prepared with Milli-Q water and membrane (0.2 μm, Whatman) filtered.

Supporting Methods

1. The plasmid DNAs. The gene coding for M2 MspA[1], MspA-C[2], MspA-D[2], MspA-H[2], MspA-M[3] were custom synthesized **(Table S2)** and respectively constructed in pET-30a(+) plasmid DNAs (Genscript, New Jersey). A hexa-histidine tag at the C-terminus of each gene was designed for nickle affinity purifications. The C, D, H or M character in the name of each plasmid respectively stands for the cysteine, the aspartic acid, the histidine or the methionine intentionally placed at site 91. All plasmid DNAs discussed in this manuscript are shared in the molecular cloud plasmid repository (GenScript, New Jersey). The access codes are MC_0101191 (M2 MspA), MC_0068403 (MspA-C), MC_0068403 (MspA-D), MC_0068402 (MspA-H) and MC_0010018 (MspA-M). Citation is requested when publishing with any of these plasmids.

2. Preparation of M2 MspA. As a proof of principle test, the nanopore preparation protocol was first applied to prepare M2 MspA. 100 ng plasmid DNA encoding for M2 MspA was heat-shock transformed into 100 μL *E.coli* BL21 (DE3) competent cells (Biomed). The competent cells and plasmid DNA were mixed in a 1.5 mL tube and gently tapped. Then the tube was ice incubated for 30 min. The tube was then incubated at 42 ℃ for 90 s in a metal heating block and ice incubated for another 3 min. The transformed cells were spread evenly on the surface of an agar plate (0.76 g LB agar with 20 mL Milli-Q water for each plate) and incubated at 37 ℃ for 18 h.

A single colony was picked up and added to a 50 mL tube containing 15 mL LB (Luria-Bertani) medium with 3 mg/mL kanamycin sulfate added. The tube was shaken (175 rpm) at 37 ℃ for 5 h till OD_{600}=0.7. It is then inducted by 0.1 mM isopropyl β-D-thiogalactoside (IPTG) and shaken (175 rpm) at 16 ℃ overnight. Afterwards, the cells were harvested by centrifugation (4000 rpm, 20 ℃, 20 min). The collected bacterial pellet (~ 300 μL) was resuspended in a 3 mL lysis buffer (100 mM Na_2HPO_4/NaH_2PO_4, 0.1 mM EDTA, 150 mM NaCl, 0.5% (w/v) Genapol X-80, pH=6.5), heated at 90 ℃ for 10 min and then centrifuged (13000 rpm, 4 ℃, 40 min).

The supernatant of the bacterial lysate was collected and added to the pre-treated beads **(Methods 4)** and shaken on a rotary mixer at room temperature (RT) for 60 min. After magnetic separation, the supernatant was discarded. Then 1 mL washing buffer A (0.5 M NaCl, 20 mM HEPES, 5 mM

imidazole, 0.5% (w/v) Genapol X-80, pH=8.0) was added to the beads. The beads were resuspended and shaken on a rotary mixer at room temperature (RT) for 5 min. After magnetic separation, the supernatant was discarded. Then 100 μL eluting buffer B1 (0.5 M NaCl, 20 mM HEPES, 219.5 mM imidazole, 0.5% (w/v) Genapol X-80, pH=8.0) was added to the beads. The beads were resuspended and shaken on a rotary mixer at room temperature (RT) for another 5 min. After magnetic separation, the supernatant was discarded. Eventually, 100 μL eluting buffer B2 (0.5 M NaCl, 20 mM HEPES, 500 mM imidazole, 0.5% (w/v) Genapol X-80, pH=8.0) was added to the beads. The tube was shaken on a rotary mixer at room temperature (RT) for 5 min. After magnetic separation, the supernatant was collected. Based on results of gel electrophoresis characterization **(Figure S3A)**, the collected supernatant contains the desired M2 MspA in an octameric form, ready for all downstream nanopore measurements without any further purifications.

3. Electrophysiology recordings and data analysis. All electrophysiology measurements were performed as previously reported[4]. Briefly, a measurement chamber is consisted of two compartments separated by a Teflon film (30 μm thick) with an orifice (~100 μm in diameter). Before the measurement, the compartments were first cleaned with Milli-Q water and ethanol. They were then blown dry with compressed nitrogen gas. The orifice on the film was pretreated with pentane containing 0.5% (v/v) hexadecane and air dried to evaporate the remaining solvent. The measurement device was placed in a Faraday cage, which serves to shield external electromagnetic noises. The Faraday cage was mounted on a floating table, which effectively minimizes mechanic vibrations. Prior to the measurement, approximately a 0.5 mL of electrolyte buffer was added to both compartments. A pair of Ag/AgCl electrodes were placed in each compartment, in contact with the buffer to form a closed circuit. By convention, the compartment that is electrically grounded is defined as the *cis* and the opposing side was defined as the *trans*. 100 μL 1,2-Diphytanoyl-sn-glycero-3-phosphocholine (DPhPC) was added to both compartments. To form a self-assembled phospholipid bilayer, the buffer in one of the compartments was pipetted up and down till the two compartments were electrically sealed. MspA nanopores were added to *cis* to initiate spontaneous pore insertions. To avoid further channel insertions, the buffer in the *cis* compartment was immediately exchanged with fresh buffer upon a single nanopore insertion.

All electrophysiology signals were amplified by an Axopatch 200B patch clamp amplifier and digitized by a Digidata 1550 B digital-to-analog converter (Molecular Devices, UK) with a 25 kHz sampling rate and filtered at a 1 kHz angular frequency. All single molecule sensing events were detected by the built-in “single-channel search” function of Clampfit 10.7 (Molecular Devices, UK) and further analyzed (histogram generation, fitting and plotting) by Origin Pro 2016.

4. Pre-treatment of Ni-charged magbeads. The Ni-charged magbeads (Cat. No. L00295, Genscript) were supplied in phosphate buffered saline (PBS), pH=7.4 containing 20% ethanol. To initiate the use of magbeads, transfer 100 μL beads solution to a 1.5 mL tube. Discard the supernatant after magnetic separation. Add 1 mL LE buffer (50 mM NaH_2PO_4, 300 mM NaCl, pH=8.0) to the beads and vortex for 30 seconds and discard the supernatant after magnetic separation. The beads were further washed with LE buffer for another 3 times and the LE buffer was eventually discarded. After pre-treatment, the beads are ready to be used.

5. Regeneration of Ni-charged magbeads. After each use, the magbeads can be regenerated for multiple further uses. Briefly, 400 μL cleaning buffer Ⅰ (20 mM Tris-HCl, 100 mM EDTA, pH=8.0) was added to the magbeads and vortexed for 30 min. After magnetic separation, the supernatant was discarded. Afterwards, 400 μL Milli-Q water was added to the beads and vortexed for 10-second. After magnetic separation, the supernatant was discarded. Repeat the above procedure of Milli-Q water washing for three times. Afterwards, 400 μL cleaning buffer Ⅱ (1 M NaOH, 2 M NaCl) was added to the beads and vortexed for 30 min. After magnetic separation, the supernatant was discarded. Next, wash the beads with Milli-Q water till the pH of the liquid was changed back to neutral. Then discard the supernatant. 400 μL 100 mM $NiSO_4$ was added to the beads. Mix the beads with the buffer for 30 min at room temperature on a rotary mixer. Finally, wash the beads with Milli-Q water for another 6 times. Exchange the liquid to 20% ethanol and store the re-charged Ni-charged magbeads at 4 °C. The regenerated beads can be re-used for more than five times without noticeable loss of performance.

6. Cost estimation of MspA preparation. The entire pore preparation process does not require any high-end instruments. The major cost of consumables per MspA preparation was roughly

estimated based on a 15 mL culturing volume for one type of MspA. To be specific, these consumables include 0.76 g LB agar ($ 0.04 per use), 0.12 g LB broth ($ 0.006 per use) and 100 μL magnetic beads. The magnetic beads can be reused for 5 times so the cost per single used was estimated accordingly ($ 0.34 per use). Though the actual cost may slightly vary due to different suppliers of consumables, the overall cost should be ~0.4 $ per single preparation, highly affordable by most academic groups and attractive for large scale industrial manufacturing.

7. Optical single channel recording (oSCR). oSCR was performed as previously reported[5]. Briefly, to prepare the lipid oil solution, 25 mg 1,2-Diphytanoyl-sn-glycero-3-phosphocholine (DPhPC) (Avanti Polar Lipids) was dissolved in pentane in a 5 mL glass vial. It is then air dried with compressed nitrogen gas till a thin lipid film was formed on the inner wall of the glass bottle. To prepare a 5 mg/mL lipid/oil solution, a 5 mL mixture of hexadecane and silicone oil with a 1:1 volume ratio and thoroughly vortexed to reach a homogeneous distribution. MspA nanopores, EDTA (400 μM) and Fluo-8H sodium salt (33.3 μM) were placed in the potassium chloride buffer (1.5 M KCl, 10 mM HEPES, pH=7.0), and then incubated in lipid/oil solution (5 mg/mL) to form a self-assembled lipid monolayer. Afterwards, the droplet was placed on an agarose substrate in a lipid/oil environment, forming a droplet interface bilayer (DIB).

TIRF measurements were performed with an inverted microscope (Eclipse Ti-U, Nikon) equipped with an oil immersion objective (60× Plan Apo TIRF, Nikon). The fluorescence was excited through a DPSS laser (100 mw, Changchun New Industries Optoelectronics Technology) at 473 nm and imaged by an electron-multiplying charge-coupled device (EM-CCD) camera (iXon3 897, Andor). The exposure time was set at 30 ms. The field of view is 135 μm by 135 μm. The fluorescent time traces were extracted by the Solis software (Andor, UK). The corresponding data analysis (histogram generation, fitting and plotting) was performed by Origin Pro 2016.

Table S1. Strand Sequences.

Strand	Sequence (5'-3')	Length (nt)
Template	AAA AAA CGT CAG AAT GTT AGA ATG TTA GAA TGT TAG AAT GTT AGA ATG TTA GAA TGT TTC AGA TCT CAC TAT CGC ATT CTC ATG CAG GTC GTA GCC	96
Primer	GGC TAC GAC CTG CAT GAG AAT GC	23
Blocker	GAT AGT GAG ATC TGA TTT CCC AAA TTT AAA /cholesterol /	30

Table S2. Protein sequences of MspA mutants.

Name	Protein Sequence
M2 MspA	MGLDNELSLVDGQDRTLTVQQWDTFLNGVFPLDRNRLTREWFHSGRAK YIVAGPGADEFEGTLELGYQIGFPWSLGVGINFSYTTPNILINNGNITAPPF GLNSVITPNLFPGVSISARLGNGPGIQEVATFSVRVSGAKGGVAVSNAHGT VTGAAGGVLLRPFARLIASTGDSVTTYGEPWNMNHHHHHH*
MspA-C	MGLDNELSLVDGQDRTLTVQQWDTFLNGVFPLDRNRLTREWFHSGRAK YIVAGPGADEFEGTLELGYQIGFPWSLGVGINFSYTTPNILICNGNITAPPFG LNSVITPNLFPGVSISARLGNGPGIQEVATFSVRVSGAKGGVAVSNAHGTV TGAAGGVLLRPFARLIASTGDSVTTYGEPWNMNHHHHHH*
MspA-D	MGLDNELSLVDGQDRTLTVQQWDTFLNGVFPLDRNRLTREWFHSGRAK YIVAGPGADEFEGTLELGYQIGFPWSLGVGINFSYTTPNILIDNGNITAPPF GLNSVITPNLFPGVSISARLGNGPGIQEVATFSVRVSGAKGGVAVSNAHGT VTGAAGGVLLRPFARLIASTGDSVTTYGEPWNMNHHHHHH*
MspA-H	MGLDNELSLVDGQDRTLTVQQWDTFLNGVFPLDRNRLTREWFHSGRAK YIVAGPGADEFEGTLELGYQIGFPWSLGVGINFSYTTPNILIHNGNITAPPF GLNSVITPNLFPGVSISARLGNGPGIQEVATFSVRVSGAKGGVAVSNAHGT VTGAAGGVLLRPFARLIASTGDSVTTYGEPWNMNHHHHHH*
MspA-M	MGLDNELSLVDGQDRTLTVQQWDTFLNGVFPLDRNRLTREWFHSGRAK YIVAGPGADEFEGTLELGYQIGFPWSLGVGINFSYTTPNILIMNGNITAPPF

	GLNSVITPNLFPGVSISARLGNGPGIQEVATFSVRVSGAKGGVAVSNAHGT VTGAAGGVLLRPFARLIASTGDSVTTYGEPWNMNHHHHHH*

Footnote:

1. * marks the stop codon.
2. The hexa-histidine tag placed on the C terminus of each gene is designed for nickel affinity chromatography purification.

Table S3. Statistics of blockage event measured with MspA-M. The measurements were carried out as described in **SI Methods 3**. A +100 mV voltage was continuously applied. The results reported below were respectively derived from measurements with 1 μM $HAuCl_4$ in *cis*. I_0 refers to the open pore current and I_b refers to the state when an analyte was bound to the pore. ΔI stands for the amplitude different between I_0 and I_b. $\Delta I/I_0$ stands for the percentage blocked current. The $\Delta I/I_0$ and dwell time derived from previously reported literatures[3] were as well provided for a comparison.

$\Delta I/I_0$ (%) This article	$\Delta I/I_0$ (%) Previously reported	Dwell time (ms) This article	Dwell time (ms) Previously reported
4.2	4.2	476.2	460.8

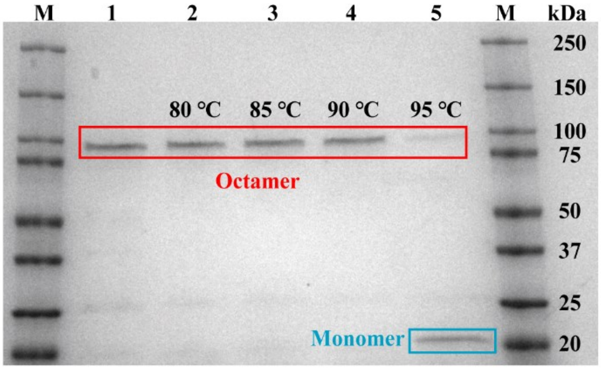

Figure S1. A thermal stability test of M2 MspA. All MspA octamers that have been tested by us demonstrate a general structural stability against high temperatures. This unique thermal stability of MspA forms the basis of this rapid and multiplex preparation method. By taking M2 MspA as a model mutant, a thermal stability test was performed for a demonstration. Briefly, a batch of previously purified M2 MspA octamers was aliquoted into different fractions. Each fraction was respectively incubated at 80 °C, 85 °C, 90 °C or 95 °C for 15 min. These fractions were then characterized by SDS gel electrophoresis as demonstrated above. Briefly, gel electrophoresis was carried out with a 4-15% Mini-PROTEAN TGX Gel (Cat. #4561083, Bio-Rad). A +200 V potential was continuously applied for 26 min. Lane M, precision plus protein standards (Bio-Rad); Lane 1, previously prepared M2 MspA octamers without thermal incubation; Lane 2, M2 MspA octamers after 15 min incubation at 80 °C; Lane 3, M2 MspA octamers after 15 min incubation at 85 °C; Lane 4, M2 MspA octamers after 15 min incubation at 90 °C; Lane 5, M2 MspA octamers after 15 min incubation at 95 °C. According to the gel results, the M2 MspA stays unchanged in an octameric form even if it has been incubated at 90 °C for 15 min. However, it is disassembled into monomers when incubated at 95 °C.

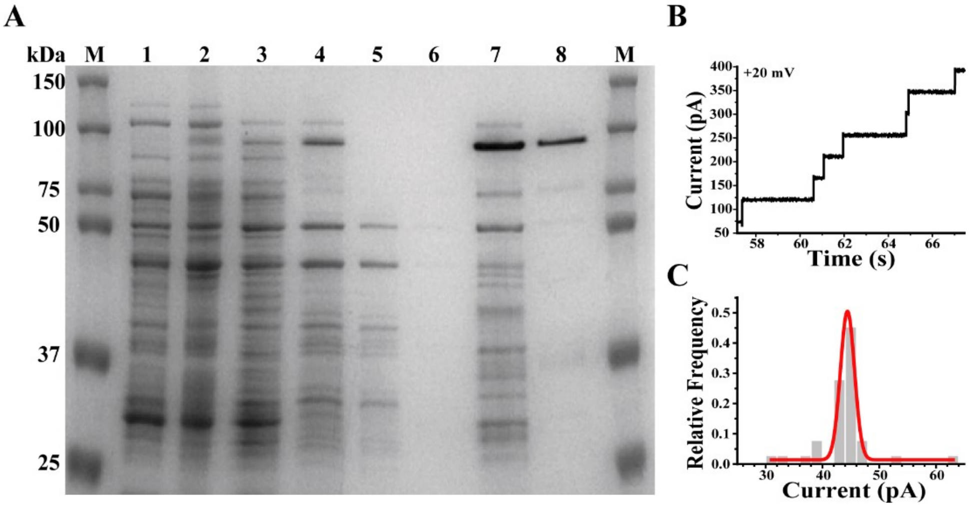

Figure S2. Characterization of M2 MspA. (**A**) Gel electrophoresis of M2 MspA. The M2 MspA were prepared as described in **Figure 1**. Gel electrophoresis was performed with a 200 V potential for 2 h on a 12% SDS-polyacrylamide gel. Lanes M, precision plus protein standards (Bio-Rad); Lane 1, total proteins of *E. coli* BL21/pET-30a(+) before induction; Lane 2, total proteins of *E. coli* BL21/pET-30a(+) after induction with 0.1 mM IPTG overnight; Lane 3, supernatant of the bacterial lysate; Lane 4, supernatant of the bacterial lysate after incubation at 90 ℃ for 10 min; Lane 5, supernatant of the bacterial lysate after incubating with Ni-charged magnetic beads; Lane 6, eluent after washing with buffer A (0.5 M NaCl, 20 mM HEPES, 5 mM imidazole, 0.5% (w/v) Genapol X-80, pH=8.0); Lane 7, eluent after washing with buffer B1 (0.5 M NaCl, 20 mM HEPES, 219.5 mM imidazole, 0.5% (w/v) Genapol X-80, pH=8.0); Lane 8, eluent after washing with buffer B2 (0.5 M NaCl, 20 mM HEPES, 500 mM Imidazole, 0.5% (w/v) Genapol X-80, pH=8.0). According to the gel results, the band ~100 kDa was identified to be the target protein, M2 MspA in an octameric form[1]. (**B**) Spontaneous insertions of M2 MspA. The measurement was performed in a 1 M KCl buffer (1M KCl, 10 mM HEPES, pH=7.0). A +20 mV voltage was continuously applied. Equally spaced current steps represent sequential insertions of M2 MspAs. (**C**) The histogram of open pore currents acquired with M2 MspA. The measurement was performed as described in **B**. Results from 40 nanopores were included to form the statistics (N=40). The distribution is overlaid with the corresponding Gaussian fitting. The M2 MspA applied for all measurements in **B, C** was from the eluent as demonstrated in the gel results of **A**.

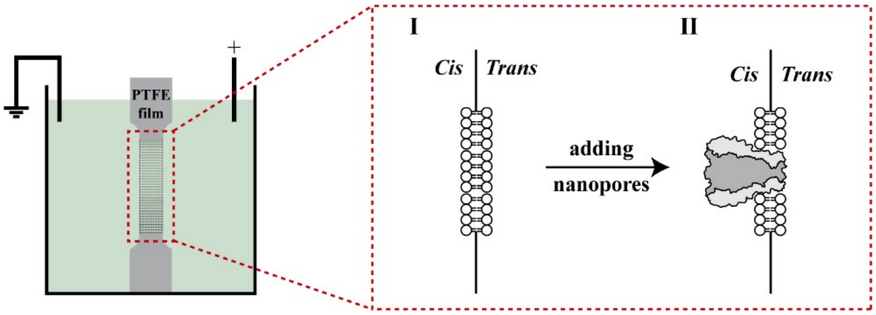

Figure S3. The diagram of single channel recording. The diagram of the measurement chamber. A measurement chamber is consisted of two compartments separated by a polytetrafluoroethylene (PTFE) film (30 μm thick) with an orifice (~100 μm in diameter). The compartment that is electrically grounded is defined as the *cis* and the opposing side was defined as the *trans*. A pair of Ag/AgCl electrodes were placed in each compartment, in contact with the buffer to form a closed circuit. 100 μL 1,2-Diphytanoyl-sn-glycero-3-phosphocholine (DPhPC) was added to both compartments to form a self-assembled phospholipid bilayer. The diagram in the dashed box describes how an MspA nanopore inserts into the lipid membrane. Ⅰ, a self-assembled phospholipid bilayer on the orifice separating the two chambers; Ⅱ, an MspA nanopore inserted in the phospholipid bilayer. All nanopore measurements in this paper were based on the above described configuration.

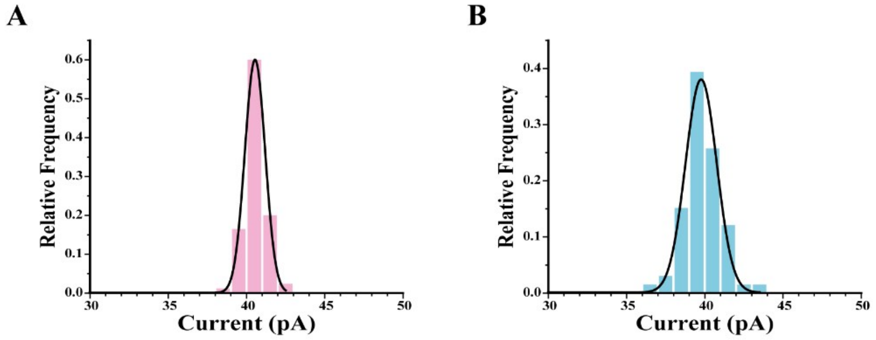

Figure S4. The histogram of open pore currents acquired with M2 MspA. (A) The histogram of open pore currents (I_0) acquired with M2 MspA prepared by the protocol described in this paper. **(B)** The histogram of open pore currents (I_0) acquired with M2 MspA prepared by the previously reported method[5]. Results in **(A, B)** were respectively acquired from 85 nanopores for each condition to form the statistics (N=85). The distributions were overlaid with corresponding Gaussian fitting results. According to the fitting results, M2 MspAs prepared by both measurements report a mean open pore current of 40 pA. Both measurements were performed in a 1 M KCl buffer (1M KCl, 10 mM HEPES, pH=7.0) and a +20 mV voltage was continuously applied. All other conditions were also kept identical.

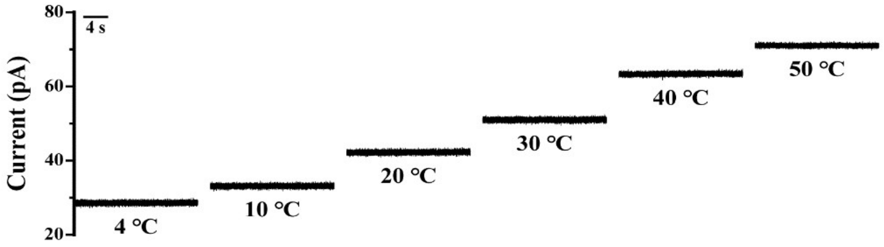

Figure S5. Open pore current of M2 MspA acquired with different temperatures. In electrophysiological measurements, MspA nanopore remains stable when the measurement temperature was set between 4 and 50 °C. The open pore current of MspA is 28.5 pA, 32.9 pA, 42.2 pA, 51.0 pA, 63.5 pA, 71.0 pA at 4 ℃, 10 ℃, 20 ℃, 30 ℃, 40 ℃, 50 ℃, respectively. The measurement was performed with an Orbit Mini apparatus (Nanion Technologies, Germany). A 1 M KCl buffer (1M KCl, 10 mM HEPES, pH=7.0) and a +20 mV voltage was continuously applied.

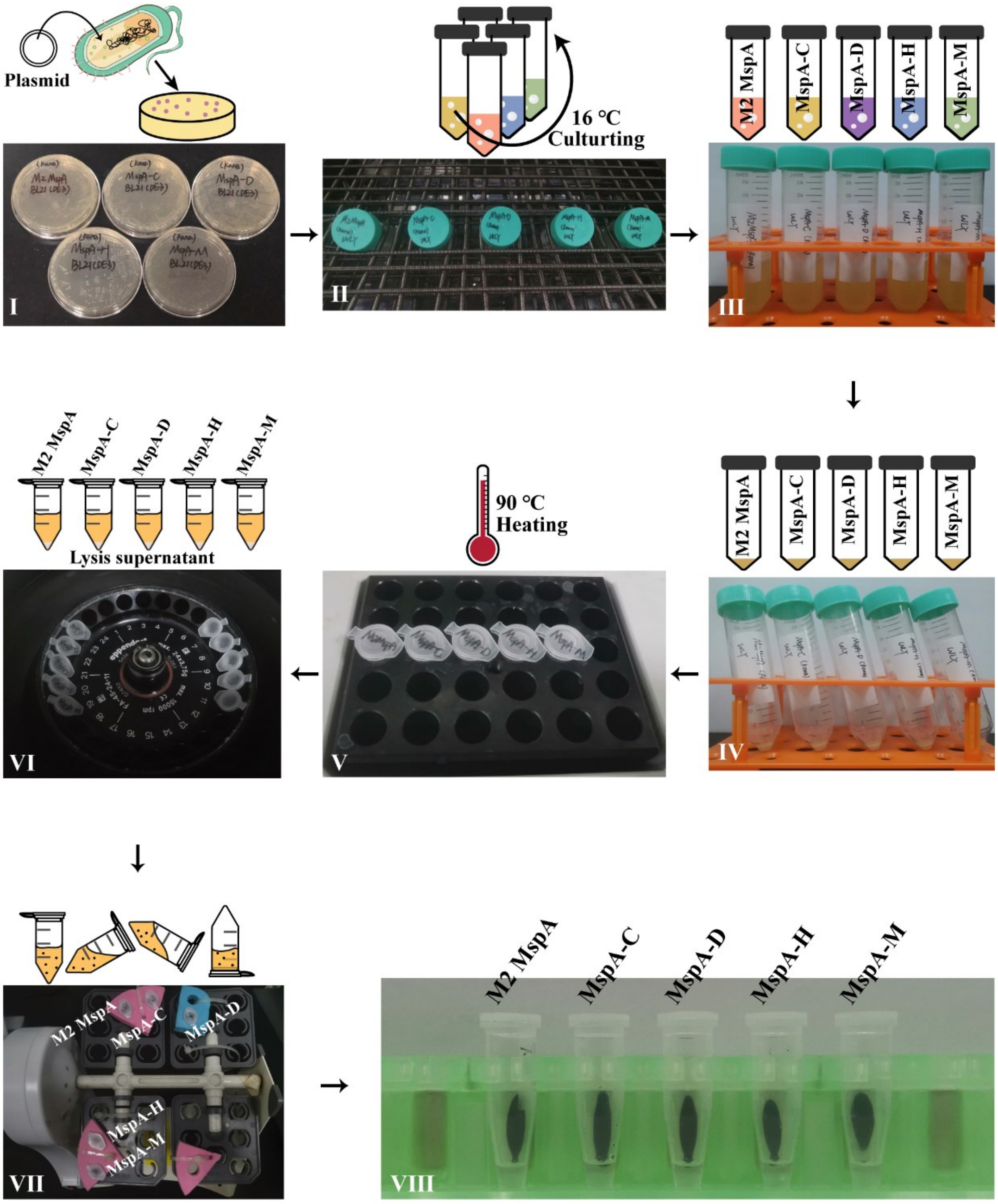

Figure S6. Multiplex preparation of five MspA mutants. Photos and schematic diagrams of the experimental operation are demonstrated. Ⅰ, Agar plates containing *E.coli* colonies; Ⅱ, Liquid cultures in a thermal incubator; Ⅲ, Liquid cultures containing grown bacterial; Ⅳ, Harvested bacterial pellets; Ⅴ, Thermal treatment of the bacterial lysate in a metal heating block; Ⅵ, Bacterial lysate centrifugation in a centrifuge; Ⅶ, Incubation with Ni-charged magbeads on a rotary mixer; Ⅷ, Multiplex magnetic separation on a magnetic separation rack. All above described operations can be routinely performed in parallel in an established molecular biology lab. No high-end instruments were required.

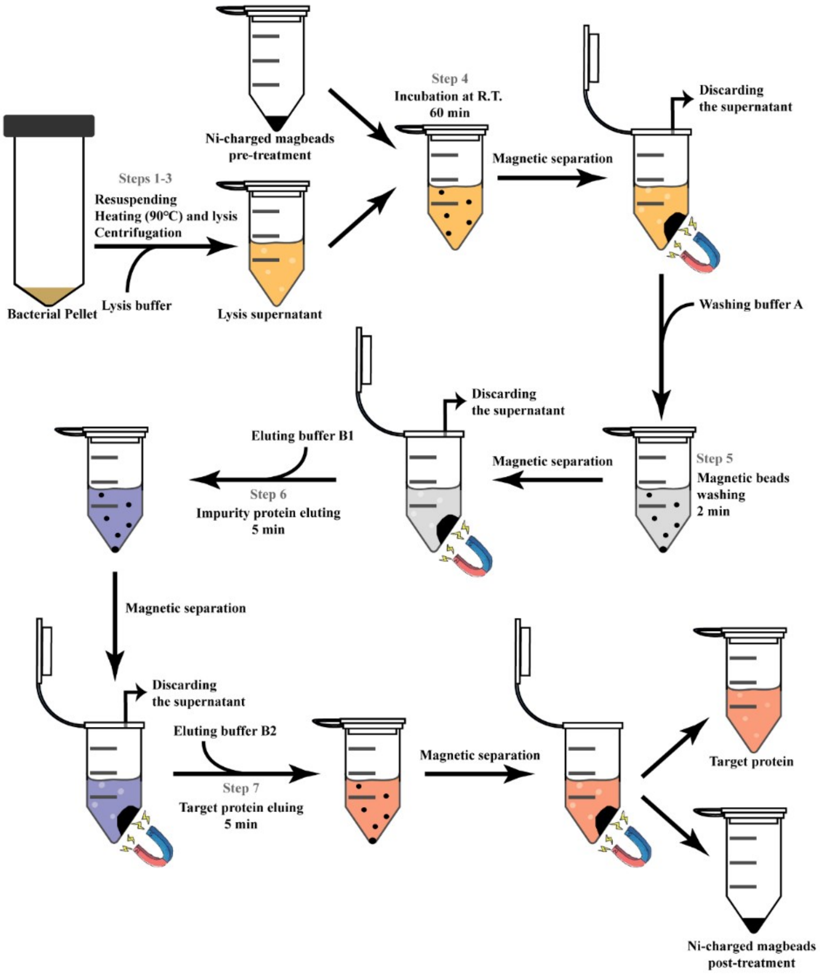

Figure S7. Rapid and multiplex purification of MspA from bacteria pellet. The bacterial pellet (~300 µL) was resuspended in a 3 mL lysis buffer **(Experimental Section)** and transformed into a 1.5 mL tube. The suspension was heated to 90 ℃ for 10 min to lyse the cells. After centrifugation, the supernatant of bacterial lysate was collected. added to pre-treated Ni-charged magbeads **(Methods 4)**. The mixture was shaken on a rotary mixer at room temperature (RT) for 60 min. After magnetic separation, the supernatant was discarded and exchanged with washing buffer A (1 mL). Resuspension was performed by shaken on a rotary mixer and set for a 5 min of incubation at room temperature (RT). After magnetic separation, the supernatant was discarded and exchanged with the eluting buffer B1 (100 µL). Resuspension was performed by shaken on a rotary mixer and set for another 5 min of incubation at room temperature (RT). After magnetic separation, the supernatant was discarded and exchanged with the eluting buffer B2 (100 µL). Resuspension was performed by shaken on a rotary mixer and set for another 5 min of incubation at room temperature (RT). After magnetic separation, the supernatant, which contains the target proteins, was collected. The Ni-charged magbeads can be regenerated for multiple times of use **(Methods 5)**.

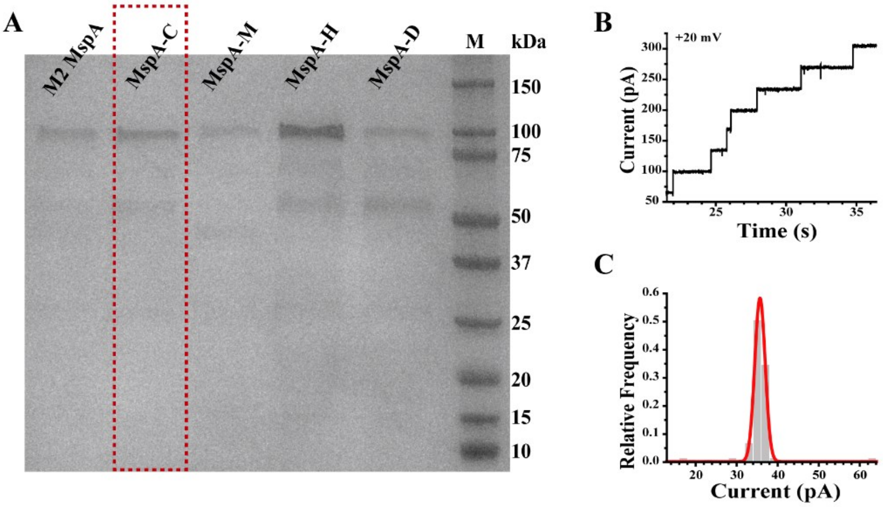

Figure S8. Characterization of MspA-C. (**A**) Gel electrophoresis results of five MspA mutants simultaneously prepared. Gel electrophoresis was carried out on a 4-20% Mini-PROTEAN TGX Gel (Cat. #4561083, Bio-Rad) and a 200 V bias was continuously applied for 27 min. The band, which is at ~100 kDa in the highlighted lane, represents the octameric MspA-C. (**B**) Spontaneous insertions of MspA-C. The batch of MspA-C, as characterized in **A**, was directly used without any further purifications. Single channel recording was performed in a 1 M NaCl buffer (1M NaCl, 10 mM HEPES, 0.4 mM TCEP, pH=7.4) and a +20 mV voltage was continuously applied. The recorded current steps represent sequential insertions from MspA-C. (**C**) The histogram of open pore currents acquired with MspA-C. The measurement was performed as described in **B**, during which a +20 mV bias was applied to evaluate the open pore current. Open pore currents from 40 nanopores were included to form the statistics (N=40). The distribution was overlaid with the corresponding Gaussian fitting results.

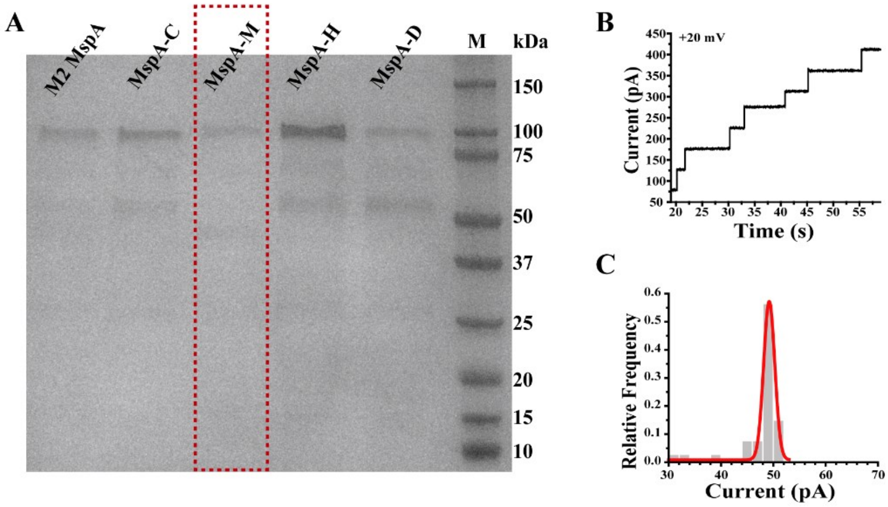

Figure S9. Characterization of MspA-M. (**A**) Gel electrophoresis results of five MspA mutants simultaneously prepared. Gel electrophoresis was carried out on a 4-20% Mini-PROTEAN TGX Gel (Cat. #4561083, Bio-Rad) and a 200 V bias was continuously applied for 27 min. The band, which is at ~100 kDa in the highlighted lane, represents the octameric MspA-M. (**B**) Spontaneous insertions of MspA-M. The batch of MspA-M, as characterized in **A**, was directly used without any further purifications. Single channel recording was performed in a 1.5 M KCl buffer (1.5 M KCl, 10 mM Tris-HCl, pH=7.0) and a +20 mV voltage was continuously applied. The recorded current steps represent sequential insertions from MspA-M. (**C**) The histogram of open pore currents acquired with MspA-M. The measurement was performed as described in **B**, during which a +20 mV bias was applied to evaluate the open pore current. Open pore currents from 41 nanopores were included to form the statistics (N=41). The distribution was overlaid with the corresponding Gaussian fitting results.

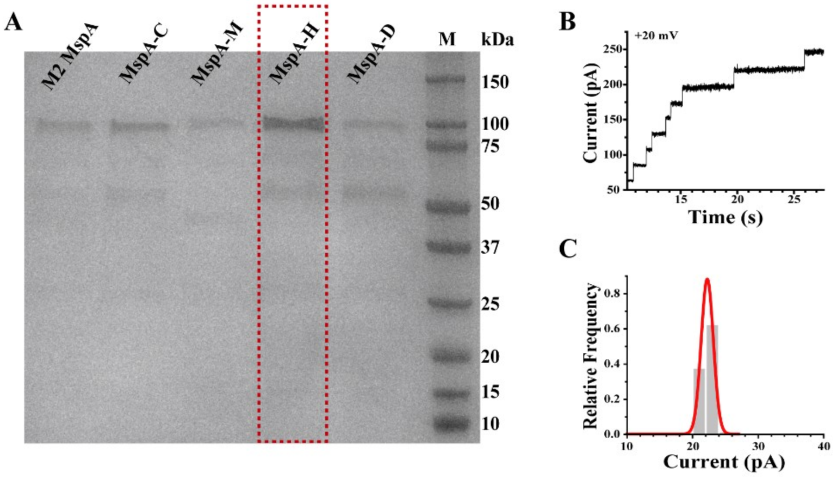

Figure S10. Characterization of MspA-H. (**A**) Gel electrophoresis results of five MspA mutants simultaneously prepared. Gel electrophoresis was carried out on a 4-20% Mini-PROTEAN TGX Gel (Cat. #4561083, Bio-Rad) and a 200 V bias was continuously applied for 27 min. The band, which is at ~100 kDa in the highlighted lane, represents the octameric MspA-H. (**B**) Spontaneous insertions of MspA-H. The batch of MspA-H, as characterized in **A**, was directly used without any further purifications. Single channel recording was performed in a 1 M NaCl buffer (1M NaCl, 10 mM HEPES, 0.4 mM TCEP, pH=7.4) and a +20 mV voltage was continuously applied. The recorded current steps represent sequential insertions from MspA-H. (**C**) The histogram of open pore currents acquired with MspA-H. The measurement was performed as described in **B**, during which a +20 mV bias was applied to evaluate the open pore current. Open pore currents from 97 nanopores were included to form the statistics (N=97). The distribution was overlaid with the corresponding Gaussian fitting results.

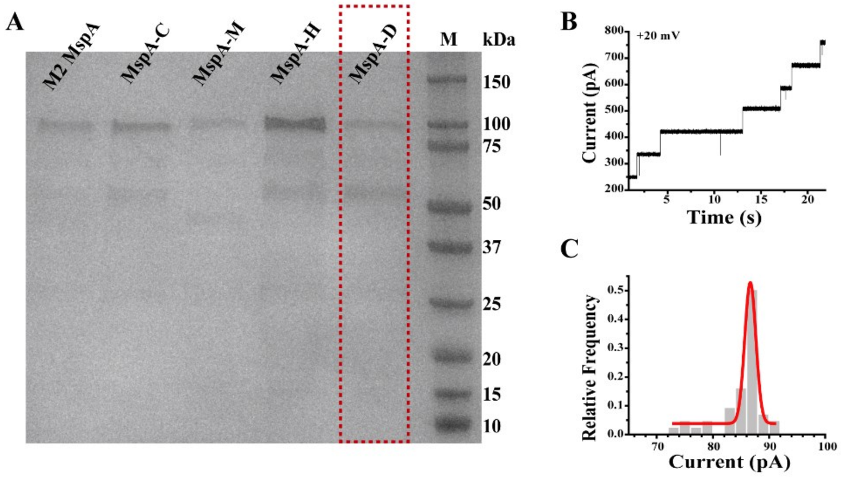

Figure S11. Characterization of MspA-D. (**A**) Gel electrophoresis results of five MspA mutants simultaneously prepared. Gel electrophoresis was carried out on a 4-20% Mini-PROTEAN TGX Gel (Cat. #4561083, Bio-Rad) and a 200 V bias was continuously applied for 27 min. The band, which is at ~100 kDa in the highlighted lane, represents the octameric MspA-D. (**B**) Spontaneous insertions of MspA-D. The batch of MspA-D, as characterized in **A**, was directly used without any further purifications. Single channel recording was performed in a 1 M NaCl buffer (1M NaCl, 10 mM HEPES, 0.4 mM TCEP, pH=7.4) and a +20 mV voltage was continuously applied. The recorded current steps represent sequential insertions from MspA-D. (**C**) The histogram of open pore currents acquired with MspA-D. The measurement was performed as described in **B**, during which a +20 mV bias was applied to evaluate the open pore current. Open pore currents from 44 nanopores were included to form the statistics (N=44). The distribution was overlaid with the corresponding Gaussian fitting results.

Video S1. Microscopic imaging of M2 MspA. Optical single channel recording (oSCR) was performed as described in the **Experimental Section**. Briefly, a droplet interface bilayer (DIB) was formed between a micro-droplet (~200 nL, 1.5 M KCl, 400 μM EDTA, 33 μM Fluo-8, 10 mM HEPES, pH=7.0) and a thin layer of hydrogel (~100 nm in thickness, 0.75 M $CaCl_2$, 10. mM HEPES, pH=7.0). According to that previously reported[5], insertions of M2 MspA results in the appearance of bright fluorescence spots due to active transport of calcium ions from the hydrogel into the droplet. A voltage protocol of a square wave (1 Hz, ±100 mV) was applied which results in synchronized modulation of the fluorescence brightness during microscopic imaging (left in the video, scale bar: 20 μm). A fluorescence-time trace (right in the video) was extracted from one of the MspA nanopores, marked with the red circle in the video. The M2 MspA nanopores applied in this assay were generated as described in this manuscript **(Figure 1)**.

References

1. T. Z. Butler, M. Pavlenok, I. M. Derrington, M. Niederweis and J. H. Gundlach, *Proc. Natl. Acad. Sci.*, 2008, **105**, 20647-20652.
2. S. Wang, J. Cao, W. Jia, W. Guo, S. Yan, Y. Wang, P. Zhang, H.-Y. Chen and S. Huang, *Chem. Sci.*, 2020, **11**, 879-887.
3. J. Cao, W. Jia, J. Zhang, X. Xu, S. Yan, Y. Wang, P. Zhang, H.-Y. Chen and S. Huang, *Nat. Commun.*, 2019, **10**, 5668-5678.
4. S. Yan, X. Li, P. Zhang, Y. Wang, H.-Y. Chen, S. Huang and H. Yu, *Chemical Science*, 2019, **10**, 3110-3117.
5. Y. Wang, S. Yan, P. Zhang, Z. Zeng, D. Zhao, J. Wang, H. Chen and S. Huang, *ACS Appl. Mater. Interfaces*, 2018, **10**, 7788-7797.
